# Evaluation of Tissue Homogenization to Support the Generation of GMP-Compliant Mesenchymal Stromal Cells from the Umbilical Cord

**DOI:** 10.1155/2016/3274054

**Published:** 2016-01-21

**Authors:** Ryan J. Emnett, Aparna Kaul, Aleksandar Babic, Vicki Geiler, Donna Regan, Gilad Gross, Salem Akel

**Affiliations:** ^1^St. Louis Cord Blood Bank/Cellular Therapy Laboratory, SSM Health Cardinal Glennon Children's Hospital, St. Louis, MO 63110, USA; ^2^Department of Pediatrics, Saint Louis University School of Medicine, St. Louis, MO 63104, USA; ^3^SSM Health St. Mary's Hospital, St. Louis, MO 63117, USA; ^4^Department of Obstetrics, Gynecology and Women's Health, Saint Louis University School of Medicine, St. Louis, MO 63104, USA

## Abstract

Recent studies have demonstrated that the umbilical cord (UC) is an excellent source of mesenchymal stromal cells (MSCs). However, current protocols for extracting and culturing UC-MSCs do not meet current good manufacturing practice (cGMP) standards, in part due to the use of xenogeneic reagents. To support the development of a cGMP-compliant method, we have examined an enzyme-free isolation method utilizing tissue homogenization (t-H) followed by culture in human platelet lysate (PL) supplemented media. The yield and viability of cells after t-H were comparable to those obtained after collagenase digestion (Col-D). Importantly, kinetic analysis of cultured cells showed logarithmic growth over 10 tested passages, although the rate of cell division was lower for t-H as compared to Col-D. This slower growth of t-H-derived cells was also reflected in their longer population doubling time. Interestingly, there was no difference in the expression of mesenchymal markers and trilineage differentiation potential of cells generated using either method. Finally, t-H-derived cells had greater clonogenic potential compared to Col-D/FBS but not Col-D/PL and were able to maintain CFU-F capacity through P7. This bench scale study demonstrates the possibility of generating therapeutic doses of good quality UC-MSCs within a reasonable length of time using t-H and PL.

## 1. Introduction

A number of studies have highlighted the potential of mesenchymal stromal cells (MSCs) in tissue regeneration, immune regulation, and potentiation of ex vivo expansion of hematopoietic stem cells (HSCs) [1–4]. Clinical applications of MSCs are mainly attributed to their low immunogenicity and ability to home to sites of pathology, differentiate into various cell types, and secrete multiple bioactive molecules capable of modulating growth of other cells like HSCs and immune cells. Traditionally, MSCs have been harvested from adult sources including bone marrow (BM) and adipose tissue. Because of the invasive cell harvest procedure and the inverse relationship between adult age and MSC growth potential [5, 6], there is a pressing need for developing alternative sources for these cells. In this regard, perinatal tissues, especially placenta and umbilical cord (UC), have become attractive sources of MSCs.

MSCs can be successfully isolated from all UC samples. In vitro expanded UC-MSCs exhibit cell surface markers, differentiation capability, and immune regulatory properties comparable to those of BM-MSCs [7–9], with the added advantage of having a higher proliferation/expansion potential (greater numbers of passages to senescence) [8–10]. Such features reflect the relatively primitive nature of the UC-MSCs compared to their adult counterpart. Additionally, UC-derived cells have not been exposed to viruses and toxins and may contain less genetic abnormalities than adult tissue-derived MSCs.

UC-MSCs have been identified and isolated from various anatomic compartments especially from UC perivascular regions and Wharton's jelly (WJ) matrix [8, 11, 12]. In the absence of a discernible demarcation between MSC regions within the UC, it is difficult to establish the region-specificity of isolated cells. Although few relevant studies have demonstrated variation in the differentiation potential between MSCs isolated from different UC regions, no significant differences were reported in growth kinetics and phenotype amongst different isolates [11]. Due to the absence of any clear cut regional delineation, many investigators have chosen to obtain cells from the entire length of the UC, which would offer better cell yield for further manipulation.

Basic methods for the generation of UC-MSC include tissue explants or collagenase-based enzymatic tissue digestion (Col-D) followed by cell culture in the presence of fetal bovine serum (FBS) to support adhesion and expansion of MSCs. Explant cultures allow migration of cells out of tissue and growth of adherent cells which reliably produce MSCs; however, initial culture takes much longer to reach confluence as compared to Col-D, and explant growth may not represent cells from all regions of the cord.

To generate UC-MSCs for potential clinical purposes, a current good manufacturing practices- (cGMP-) compliant production method needs to be developed which can be reliably performed in the absence of enzymes and FBS. The use of enzymes and FBS may complicate the cGMP process development depending on the source of the enzyme and batch-to-batch variations associated with both bioproducts [13]. While some alternatives to FBS have now been developed (e.g., human serum, platelet lysate (PL), and chemically defined media supplements), [14] there is a lack of nonenzymatic methods for cellular extraction. In this study, an enzyme-free, simple cell isolation method consisting of rapid UC tissue homogenization (t-H) using a GentleMACS Dissociator (Miltenyi Biotec) was evaluated. Additionally, growth of isolated cells and generation of MSCs were assessed in the presence of PL with the aim to support the development of a cGMP-compliant protocol for cell isolation and expansion.

## 2. Materials and Methods

### 2.1. Samples

#### 2.1.1. Umbilical Cord Tissue

This study was reviewed and approved by the SSM Health Institutional Review Board (IRB). Umbilical cords (*n* = 10) were obtained after vaginal or cesarean deliveries, drained of blood, and clamped at either end. All cords were transported in sterile phosphate buffered saline (PBS) and processed within 24 hours of collection.

#### 2.1.2. Bone Marrow

BM-mononuclear cell (BM-MNC) samples (*n* = 3) were purchased from Stem Cell Technologies Inc. and thawed as per the manufacturer's guidelines.

### 2.2. Isolation of MSCs from the Umbilical Cord

Each cord (between 12 and 30 cm long) was divided into 2 cm segments and processed to extract cells using tissue homogenization or collagenase digestion.

#### 2.2.1. Tissue Homogenization (t-H)

A representative 2 cm segment was further minced, rinsed with PBS, and placed into a MACS C-tube (Miltenyi Biotec). Prewarmed basal media (low dextrose alpha MEM, Life Technologies) was added to a final volume of 10 mL and the tissue was homogenized with the GentleMACS Dissociator (Miltenyi Biotec). Contents of the C-tube were transferred to a new 50 mL falcon tube, diluted with 3 volumes of basal media, and then filtered through a 100 *μ*m strainer.

#### 2.2.2. Collagenase Digestion (Col-D)

For enzymatic digestion, the 2 cm minced tissue was rinsed with PBS, placed into a tube containing 20 mL collagenase I (2.5 mg/mL, Life Technologies), and incubated for 2 hours at 37°C with gentle mixing every 10 min. At this enzyme concentration, a 2-hour incubation time yielded the highest recovery of viable cells (data not shown). At the end of the digestion period, the sample was diluted with basal media to a volume of 50 mL, and the supernatant transferred to a new tube through a 100 *μ*m strainer.

t-H and Col-D samples were centrifuged, and cell pellets were washed twice and resuspended in basal culture media. Isolated cells from each method were assessed microscopically (using a hemocytometer) for count and viability using trypan blue (Life Technologies).

### 2.3. Culture and Expansion of Isolated UC-MSCs

Initially isolated cells representing passage zero (P0) were plated in multiple T25 flasks at a density of 1 × 10^7^ viable cells/flask in complete media (alpha MEM with 1% penicillin/streptomycin/neomycin and 1% amphotericin B) supplemented with heat inactivated 20% FBS (HyClone) or 10% human pooled cGMP-grade PL (Compass Biomedical). After 3 days, media containing nonadherent cells was removed and adherent cells were allowed to grow in fresh media with half media changes performed every 3-4 days. Once cells reached 80–90% confluence, adherent cells were trypsinized using TrypLE (Life Technologies), counted, and replated as passage 1 (P1) at a density of 2,000 viable cells/cm^2^ in culture media supplemented with either FBS or PL. Cultures were continued for 10 passages unless cells showed consistent slow growth or signs of senescence. At the end of each passage, harvested cells were evaluated for count, viability, population doublings (PD) [9], population doubling time (PDT) [2], and cumulative count per cm of UC tissue. Different aliquots of cells from various passages were cryopreserved or prepared for cell characterization as described below.

### 2.4. Generation of MSCs from the Bone Marrow

BM-MNCs were plated at an average of 2.5 × 10^6^ cells per well (6-well dish) and cultured in complete alpha MEM supplemented with either 20% FBS or 10% PL. Growth kinetics, MSC phenotype, and differentiation potential were compared to UC-MSCs.

### 2.5. Cryopreservation

Cells were cryopreserved in a Xeno-free freezing solution containing 50% alpha MEM, 30% human AB serum (Corning), and 20% Cryosure-Dex40 (WAK-Chemie). Following trypsinization of adherent cells, 2 × 10^6^ cells were aliquoted, pelleted, and resuspended in 1 mL of cryopreservation media. Cells were transferred into cryovials and passively frozen using Mr. Frosty freezing container (ThermoFisher) overnight at −80°C before being relocated to vapor liquid nitrogen storage.

### 2.6. Colony Forming Units-Fibroblasts (CFU-F) Assay

At selected passages (1, 3, 5, and 7), 100 cells were plated in triplicate wells of a 6-well plate in corresponding media (containing FBS or PL) with half media changes performed every 3-4 days. After 10–12 days, the plates were stained with 0.5% crystal violet and the number of individual colonies (>50 cells) were counted [15].

### 2.7. Immunophenotyping of Cultured MSCs

At selected passages (2, 3, 5, 7, and 10), immunophenotyping was performed by flow cytometry using the MSC phenotyping kit (Miltenyi Biotec). The kit consists of two cocktails of antibodies: a phenotyping (FITC-CD90, PE-CD105, APC-CD73, and PerCP-CD34/CD45/CD14/CD20) and an isotype control cocktail. In separate tubes, 0.5–1.0 × 10^6^ cells were suspended in staining buffer containing 1% FBS in PBS and incubated for 10 min at 4°C with either phenotyping or isotype control antibodies. Cells were then washed and resuspended in the staining buffer. Cells were also stained with PerCP-CD31 (R&D Systems) and APC-CD146 (Miltenyi Biotec) antibodies, along with their isotype controls (R&D Systems and BD Biosciences, resp.), in a similar manner. Data was acquired on the Accuri C6 cytometer (Becton Dickinson) and at least 150,000 events were collected for each marker. Analysis was performed using the BD C6 Accuri software (BD Biosciences) to determine percentage of cells expressing specific markers.

### 2.8. Assessment of Trilineage Differentiation Potential

UC-MSCs and BM-MSCs were assessed for their ability to differentiate into osteogenic, adipogenic, and chondrogenic cells using commercially available differentiation kits (Life Technologies). Briefly, for osteogenic and adipogenic differentiation, cells from passage 3 were plated at a density of 1.5 × 10^4^ cells per well (24-well plate), in their respective proliferation media. After 24 hours, differentiation was induced while control wells were continued in culture without induction. Cultures were monitored for differentiation and assayed after 21 days by Alizarin Red staining for osteogenesis or 14 days by Oil Red O staining for adipogenesis.

For chondrogenic differentiation, a micromass culture assay was performed following the manufacturer's instructions. After 18–21 days, chondrogenic pellets were fixed, paraffin-embedded, sectioned, and stained with Alcian Blue to assess chondrogenic differentiation. All photomicrographs were taken using the 10x objective of a Zeiss Axio Observer A1 inverted microscope and images were captured using the AxioVision (version 4.8.2) software (Zeiss).

### 2.9. Statistics

All data in this study is expressed as mean ± SD. Analysis was performed using GraphPad Prism (version 5.0) software using a two-tailed Student's *t*-test. Statistical significance was set at *p* < 0.05.

## 3. Results

### 3.1. Isolation of UC-MSCs: t-H versus Col-D

In order to disrupt the UC matrix and release the stromal cells, simple t-H using the GentleMACS Dissociator was performed on 10 different UC samples. When compared to the standard method of Col-D, the t-H-based isolation method had a shorter processing time (approximately 3 hours versus 1 hour). Neither the cell number per cm of tissue nor cell viability was significantly different using both isolation methods. Viable cell count/cm was 2.04 ± 1.57 × 10^7^ versus 2.69 ± 2.51 × 10^7^ and cell viability was 95.44 ± 5.17% versus 96.98 ± 5.86% for t-H versus Col-D, respectively.

Freshly isolated cells, for three samples, were screened for expression of MSC surface markers. As reported in other tissue sources [16], a very small fraction of isolated cells (<2%), regardless of the isolation method, were positive for CD90, CD105, and CD73 markers (data not shown).

**Figure 1:**
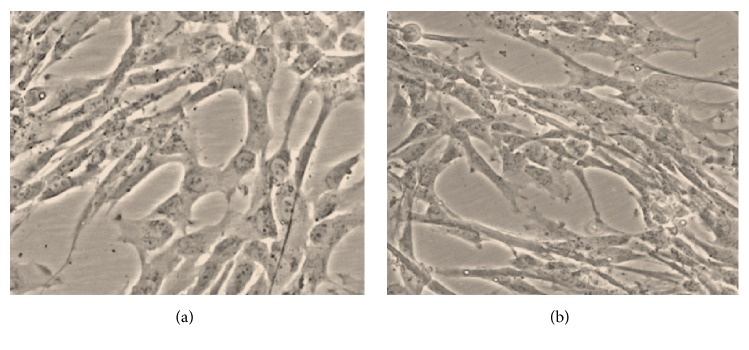
Umbilical cord MSCs are plastic-adherent with fibroblast-like morphology. Representative phase-contrast images of cultured cells generated using (a) collagenase digestion or (b) homogenization of umbilical cord tissue.

Cells released by both methods were compared for their ability to form MSCs in media supplemented with FBS and PL. Both methods generated an adherent monolayer with fibroblast-like morphology (Figure 1), characteristic of MSCs grown on a plastic culture surface. However, the time to reach 80–90% confluent growth varied among different culture conditions. Cultures of Col-D/FBS or PL reached 80–90% confluent growth within a relatively short time (8.88 ± 3.44 days) when compared to cultures of t-H/PL (18.13 ± 6.83 days). Additionally, adherent cells at different passages were verified for MSC identity by flow cytometry using a panel of surface markers suggested for defining MSCs [17] (see results in Section 3.4).

Under t-H/FBS culture conditions, majority of cases showed very slow growth and failure of expansion beyond P5 (data not shown); therefore, this condition (t-H/FBS) was less than adequate for UC-MSC production and it was excluded from our analysis.

### 3.2. Growth Characteristics and Expansion Potential of Isolated Cells

**Figure 2:**
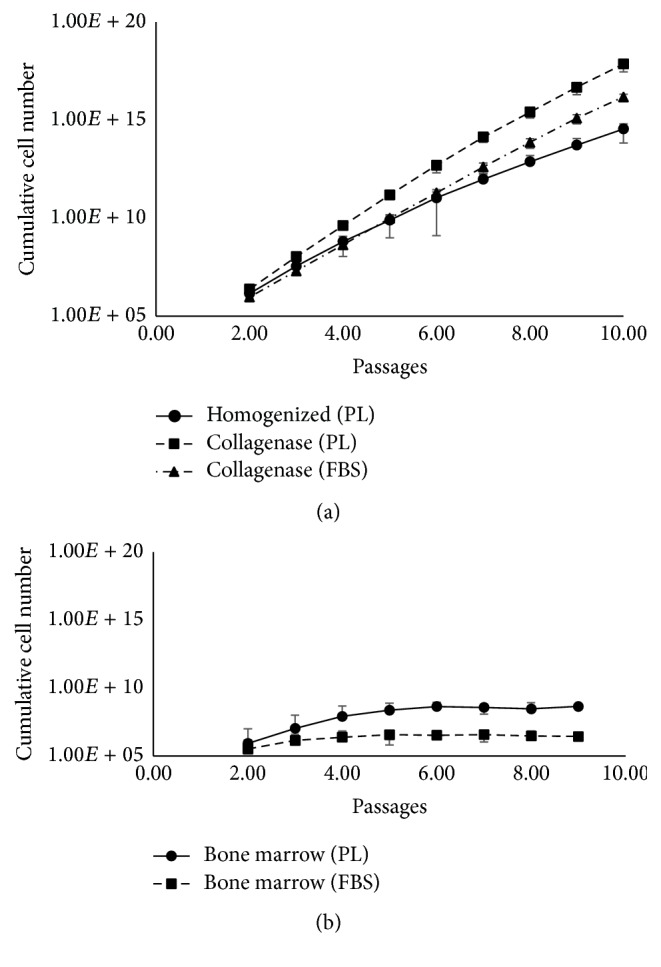
Cultured MSCs demonstrate logarithmic growth potential. Each graph depicts logarithmic growth potential and the theoretical number of cells that can be obtained using different isolation methods and culture conditions over several passages for (a) umbilical cord (*n* = 8), and (b) bone marrow (*n* = 3). Equal numbers of cells were plated for each condition and fold changes were determined after each passage (P2–P10) to determine the theoretical cell number that could be obtained starting with 50,000 cells from P1. Error bars denote mean ± SD.

In order to generate clinically relevant numbers of MSCs for therapeutic applications, it is imperative that cells have the potential of logarithmic growth and the ability to significantly expand in culture. Adherent cells generated from the above experiments were trypsinized (P1), seeded at the same density, and evaluated for growth kinetics, PD, and PDT over another 9 passages (P2–P10). For a more informative evaluation, growth of UC-MSCs was compared to the growth of BM-MSCs. In line with previous reports [2, 18, 19], successful logarithmic growth of MSCs was observed over tested 10 passages in all cultures of Col-D samples in FBS and PL. However, 8 out of 10 t-H/PL cultures showed successful logarithmic growth through P10 while a very slow growth was observed beyond P7 in cultures of the other 2 samples. As shown in Figure 2(a), the respective kinetic data of all successful cultures demonstrated logarithmic cell growth of UC-MSCs and high expansion potential. Indeed, UC-MSCs could expand through P10 without loss of proliferative activity unlike BM-MSCs where cells showed diminished replicative capacity beyond passage 5 (Figure 2(b)). This agrees with previous studies [2, 7, 20] suggesting superior proliferative potential of UC-MSCs versus BM-MSCs.

**Figure 3:**
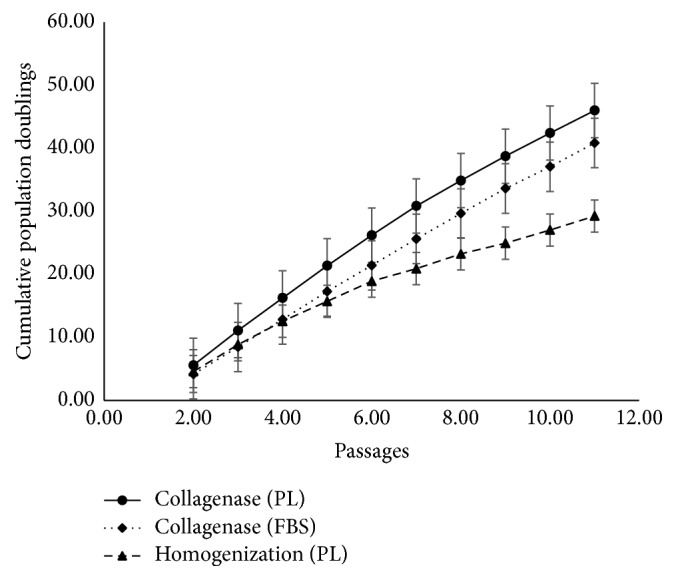
UC-MSCs continue to expand robustly in culture at the end of 10 passages. Cumulative population doublings achieved over the duration of culture demonstrate the replicative capacity of UC-MSCs generated using Col-D or t-H. Error bars denote mean ± SD.

At the end of each passage, we calculated cumulative cell number that could theoretically be generated starting with one cm of UC. By the end of P10, calculated cell numbers exceeded 1 × 10^15^ MSCs/cm under all culture conditions (Col-D/FBS, Col-D/PL, and t-H/PL). However, the cell number attained at P10 in Col-D/PL cultures (6.9 × 10^20^) was significantly higher than t-H/PL cultures (5.34 × 10^19^) corresponding to cumulative PD of 45.60 and 29.25, respectively (Figure 3). This relatively slower growth of cells from t-H was also reflected by longer mean PDT estimated for early (P1–P3) and late (P7–P9) passages (Table 1). As expected, under all culture conditions, the PDT increased for late passages indicating a reduction in the proliferative potential of cultured MSCs.

Given the above growth kinetics data, we also analyzed the number of passages and time needed to generate one billion cells from one centimeter of cord tissue. In this regard, using either t-H or Col-D and culture in PL, over a billion cells could be generated over 3 passages; however, the time to achieve this expansion varied between isolation methods (33.27 ± 7.00 days for t-H versus 22.27 ± 4.00 days for Col-D). This data is in agreement with the observation that Col-D cultures reach their first passage faster and once in culture have a shorter PDT. Collectively, this data suggests that although Col-D generates UC-MSCs faster, it is feasible to generate therapeutic doses of such cells using a cGMP-compliant protocol of t-H/PL within a reasonable length of time. Based on data obtained from the 8 cord samples using t-H/PL, a theoretical yield of 3.0 × 10^10^ UC-MSCs can be generated within approximately 1 month from a single cord of thirty cm length. This yield would allow treatment of forty patients (based on average patient weight of 70 kg and infusion of double doses each dose of 5 million cells/kg) in a phase-I clinical trial.

### 3.3. CFU-F Contents of MSC Preparations

**Figure 4:**
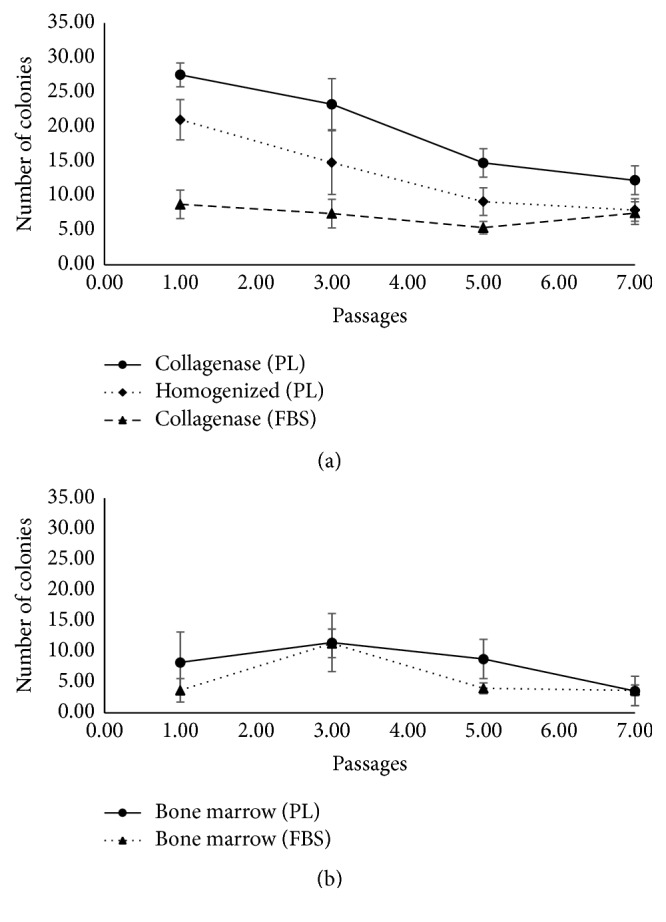
Umbilical cord MSCs possess robust self-renewal capability. Graphical representation of mean CFU-F content of MSCs, at different passages, generated by plating 100 cells per well from (a) umbilical cord and (b) bone marrow. Error bars denote mean ± SD.

The ability of MSCs to form colonies is considered an important parameter for judging the quality of cultured cells [21]. As the passage number increases, clonogenic potential of cells is expected to decrease [22]. Hence, we evaluated the clonogenic potential of MSCs generated using t-H and Col-D. In agreement with established biological characteristics of MSCs, the number of CFU-F colonies for all conditions decreased with increasing passages (Figures 4(a) and 4(b)). The CFU-F content was higher in cultures supplemented with PL compared to FBS, and UC-MSCs had superior colony forming contents as compared to BM-derived MSCs (Figure 4(b)). Importantly, t-H/PL cultures had a greater colony forming capacity compared to the conventional culture conditions of Col-D/FBS. This indicates that cells generated under cGMP-compliant conditions have a good self-renewal capacity when compared with currently used conventional protocols.

### 3.4. Phenotypic Characterization and Multilineage Differentiation Potential of UC-MSCs

**Figure 5:**
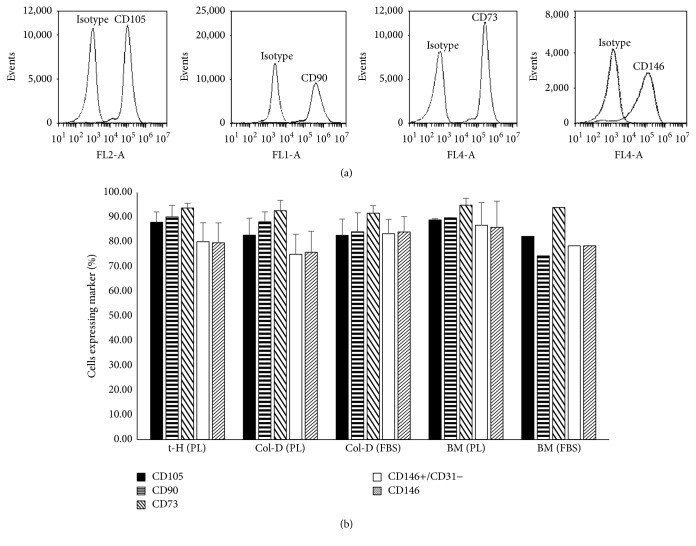
Umbilical cord MSCs express characteristic mesenchymal cell surface markers by flow cytometry. (a) Representative histograms from passage 3 demonstrate expression of mesenchymal markers (CD90, CD105, CD73, and CD146) in cultured UC-MSCs. Histograms show antibodies with their respective isotype controls. (b) Bar graph demonstrates the mean expression of different mesenchymal markers by cultured MSCs derived from umbilical cord (*n* = 8). CD45, CD34, CD14, CD20, and CD31 are not shown since expression levels were less than 2%. Bone marrow MSCs were used as controls for cell marker expression. Each bar represents mean ± SD.

**Figure 6:**
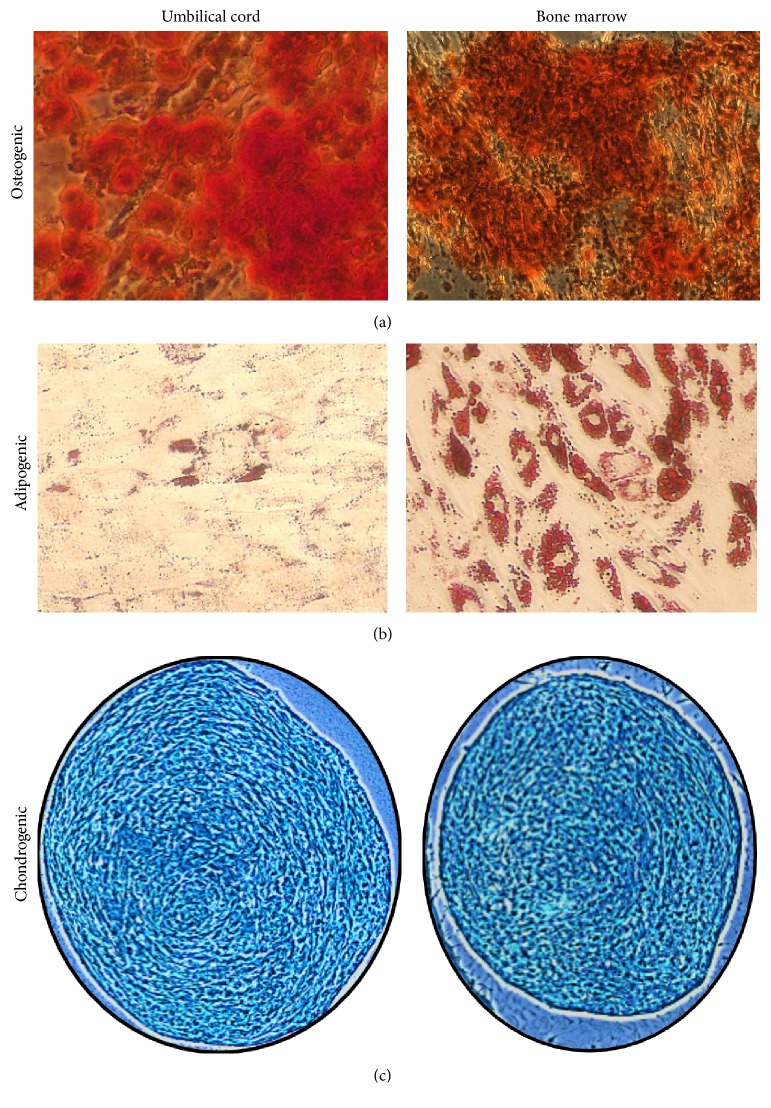
Umbilical cord MSCs exhibit trilineage differentiation potential. Representative images from passage 3 demonstrate (a) osteogenic differentiation using Alizarin Red staining, (b) adipogenic differentiation using Oil Red O, and (c) chondrogenic differentiation using Alcian Blue staining of chondrogenic pellets generated via micromass cultures. Bone marrow MSCs were used as positive control for demonstrating trilineage differentiation.

To demonstrate that cultures established from UC cells isolated by both methods are MSCs, cells were evaluated by flow cytometry in reference to criteria set forth by the ISCT [17]. In general, more than 80% of cells were found to express mesenchymal markers (CD73, CD90, and CD105) (Figure 5) and were negative (<1%) for hematopoietic (CD45, CD34), monocyte (CD14), B cell (CD20), and endothelial (CD34, CD31) markers. Moreover, UC-MSCs were evaluated for a recently identified phenotype of pericyte-like MSCs (CD146^+^CD31^−^CD34^−^CD45^−^) [23]. This population represented >75% of UC-MSCs. For both isolation methods, all mesenchymal markers were maintained over long-term culture and did not show any significant change over 10 passages. Additionally, these cells exhibited trilineage mesodermal differentiation (adipogenic, chondrogenic, and osteogenic) potential, a characteristic feature of MSCs (Figure 6). In our analysis, the differentiation potential of UC-MSCs evaluated at P3 appeared comparable in all tested samples irrespective of the isolation method and culture conditions. BM-MSCs were used as a positive control for all differentiation experiments. In agreement with previous reports, the ability of UC-MSCs to differentiate into adipocytes was extremely low when compared to BM-MSCs [20, 24].

## 4. Discussion

Traditionally, isolation of UC stromal cells has been achieved using explant cultures or through collagenase digestion of intracellular fibers. Several methodological variations have been reported (reviewed in [25]) ranging from removal of blood vessels to keeping them intact, using a combination of enzymatic digestion and explant cultures, employing collagenase alone or a combination of collagenase, hyaluronidase, and trypsin. While enzymatic digestion results in a more uniform release of cells, it has the drawbacks of longer initial processing time (anywhere from 3 to 18 hours), lack of a standard method, and being non-cGMP-compliant since the reagents are obtained from nonhuman sources making it difficult to obtain clinical-grade material. Alternatively, the explant cultures have shorter processing times but they suffer from the nonuniformity of cell migration and improper adherence of tissue fragments to the culture dish resulting in variability in the quantity and quality of cells obtained in culture [26]. Given the compartmental heterogeneity of stem cell distribution within the UC tissue [19], differential cell release may impact the “stemness” or differentiation capability of the generated MSCs [9]. Therefore, we sought to evaluate, at bench scale, a method that would result in the fast and uniform release of cells with the added advantage of using Xeno-free reagents to support the development of a cGMP-compliant protocol.

Previous reports have indicated that collagen is amenable to simple mechanical disruption [27, 28]. Based on this observation, we hypothesized that viable cells could be potentially released from the UC tissue using simple t-H in a prewarmed buffer. Therefore, we used the GentleMACS Dissociator to achieve rapid, uniform, and mild disruption of the UC matrix to release the stromal cells. To simplify the protocol and achieve better isolation yield from various tissue compartments, we chose not to strip blood vessels and amnion prior to cord homogenization. This minimal processing did not impact the purity of MSCs generated, as evidenced by the extremely low percentage of endothelial (CD31) and hematopoietic (CD45) cells in our cultures. Our protocol is based on simply cutting and mincing the entire cord into small pieces, washing to remove blood clots, and homogenizing the tissue for uniform release of cells. Furthermore, to develop and characterize a cGMP-compliant method for generation of MSCs, cell growth and expansion were tested in human PL, an accepted cGMP-compliant alternative to FBS [9, 29, 30]. In this study, we have demonstrated the possibility of producing large numbers of viable UC-MSCs using simple t-H and expansion in PL-supplemented media. Herein, we have furthered the observations on not only the feasibility but the superiority of pooled PL as a cGMP growth supplement for culturing MSCs [14, 29, 30]. In all cultures, irrespective of cell source and isolation method, PL promoted better growth of MSC when compared to FBS.

The method described here has several advantages including uniform release of cells, shorter processing time, and the absence of any enzyme of bacterial origin. The MSCs generated using t-H demonstrated exponential growth potential, excellent clonogenic capacity, and characteristic mesenchymal phenotype. Although the t-H- derived cells were slower growing when compared to Col-D, they maintained good CFU-F potential even at later passages (through P7). This is in contrast to reports demonstrating diminished or absent clonogenic capacity (beyond passage 5) of cells generated using explant cultures [9]. Furthermore, for clinical purposes, the proposed optimal time for harvesting cells is before passage 5 or 30 population doublings [31]. Using t-H, we can theoretically achieve clinically relevant number of cells in three passages and less than 10 population doublings from one cord, making this method feasible for clinical development. While the collagenase-based method generated more MSCs per cm of the cord in a shorter duration of time, there was no significant difference in terms of cellular phenotype and differentiation potential of the cells derived using t-H. Given the regulatory hurdles associated with the use of reagents of animal origin, tissue homogenization followed by growth in PL represents a simple and practical alternative to the currently used protocols. Our isolation/expansion technique utilizes GMP compatible reagents and could be further adapted into a fully GMP compliant method where other requirements shall be fulfilled such as selection of clinically eligible donors screened for infectious diseases and the assurance of product safety indicated by the absence of bacterial/fungal and mycoplasma contamination, acceptable endotoxin levels, and evidence of the lack of tumorigenicity.

Finally, we have cryopreserved cells from different isolation and culture conditions (t-H/PL, Col-D/PL, and Col-D/FBS) at various passages to generate a master bank of research cells for future studies. As mentioned in the Methods, Xeno-free reagents were used for cryopreservation so as to make this protocol cGMP-compatible at every step of the process. Initial findings after direct thaw of cells frozen for more than six months showed cell recovery and viability above 80%. In parallel, no apparent change in growth kinetic and colony formation potential was observed after thaw (data not shown). Future studies might aim toward extensive characterization of cryostored cells for stability, differentiation potential to nonmesodermal cell types, and assessment for clinically relevant indications like immunosuppression and hematopoietic support capability.

## 5. Conclusions

This study describes a bench-scale, nonenzymatic, cGMP-compatible method using whole cord tissue homogenization followed by growth in platelet lysate as an alternative to the currently used methods for generating UC-MSCs. Using this approach, clinically relevant numbers of cells, which are clonogenic, fulfil the standard criteria of MSC marker expression and mesodermal trilineage differential potential can be efficiently obtained for therapeutic applications.

## Figures and Tables

**Table 1:** Population doubling time for early and late passages.

Culture condition	Doubling time (hours) P1–P3	Doubling time (hours) P7–P9
Homogenization (PL)	41.43 ± 14.68	71.91 ± 31.70
Collagenase (PL)	30.38 ± 2.70	43.64 ± 10.14
Collagenase (FBS)	38.97 ± 6.35	44.48 ± 6.14
